# Supplementary figures and images for: STING pathway contributes to Steroid-Hyporesponsive Lung Inflammation in DSS-induced colitis mice model
Source: PLoS One. 2026 Mar 27;21(3):e0344511. doi: 10.1371/journal.pone.0344511 (PMC13028359; doi:10.1371/journal.pone.0344511)

**Supplementary Fig. 1**

**A.**

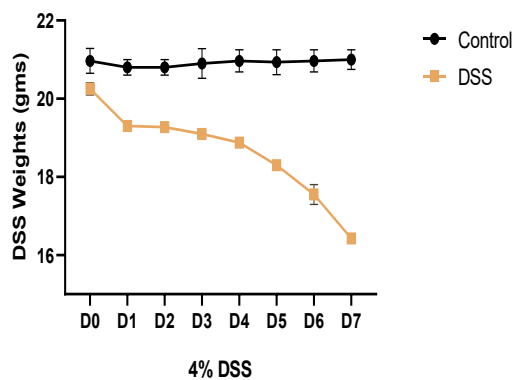

**B.**

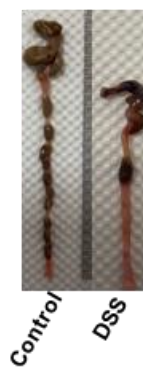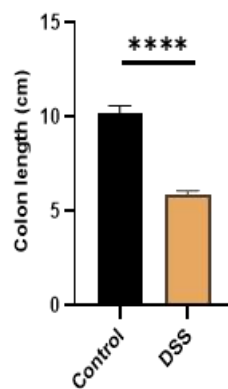

**C.**

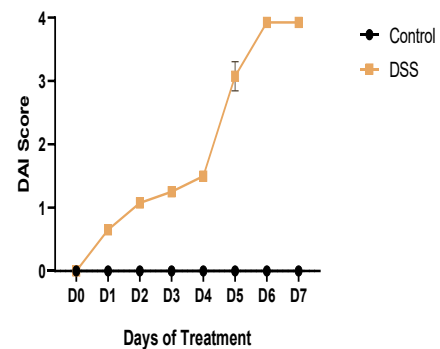

**D.**

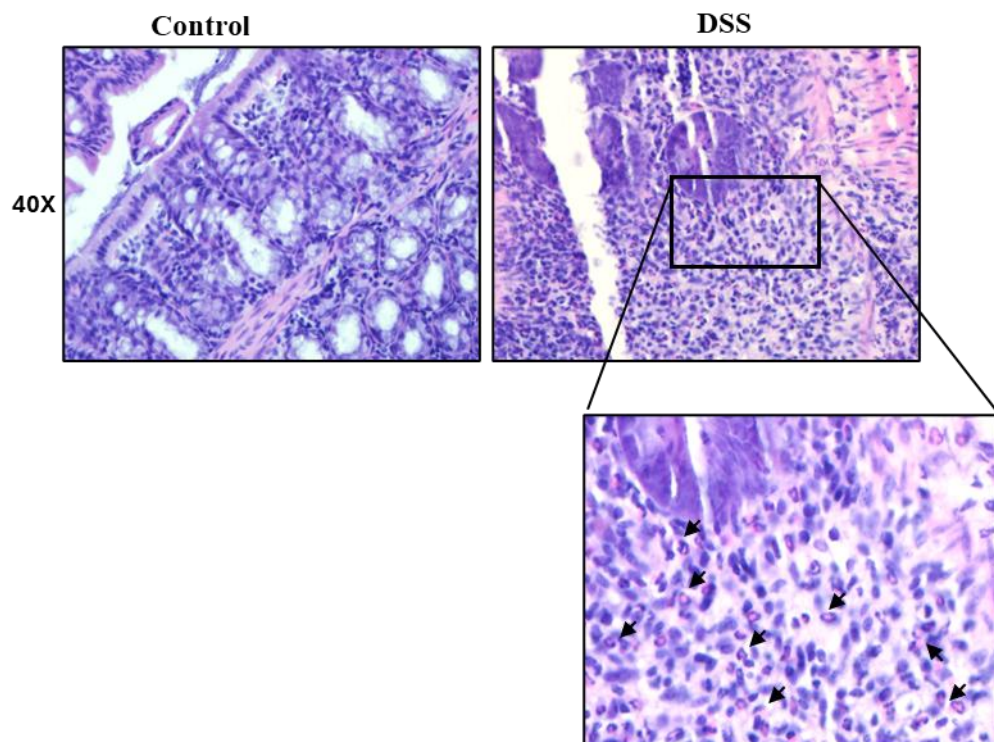

**E.**

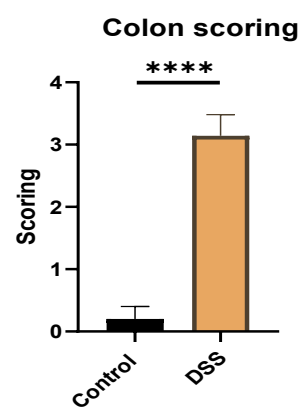

Supplement: S1 Fig — Mice were treated with 4% DSS in drinking water for 5 days, followed by 2 days of regular water, to induce acute colitis. (A) Body weight loss was observed in DSS-treated mice starting from day 4 and progressively worsened by day 7. (B) Colon length was significantly shorter in DSS-treated mice compared to controls. (C) Disease Activity Index (DAI) scores indicated an increase in colitis severity in DSS-treated mice. (D-E) Histological scoring of colonic sections showed severe colonic inflammation, with transmural infiltration, crypt damage, and disrupted epithelial and mucosal structures in DSS-treated mice compared to controls. Samples were obtained from control mice and DSS treated mice. Data represent n = 5 mice per group. Results are presented as mean ± SEM, with statistical analysis performed by one-way ANOVA followed by a post hoc Bonferroni test for multiple comparisons; *P < 0.05, **P < 0.01, ***P < 0.001, **** P < 0.0001 compared to control mice. (PDF) [file pone.0344511.s001.pdf]

**Supplementary Fig. 2**

**A.**

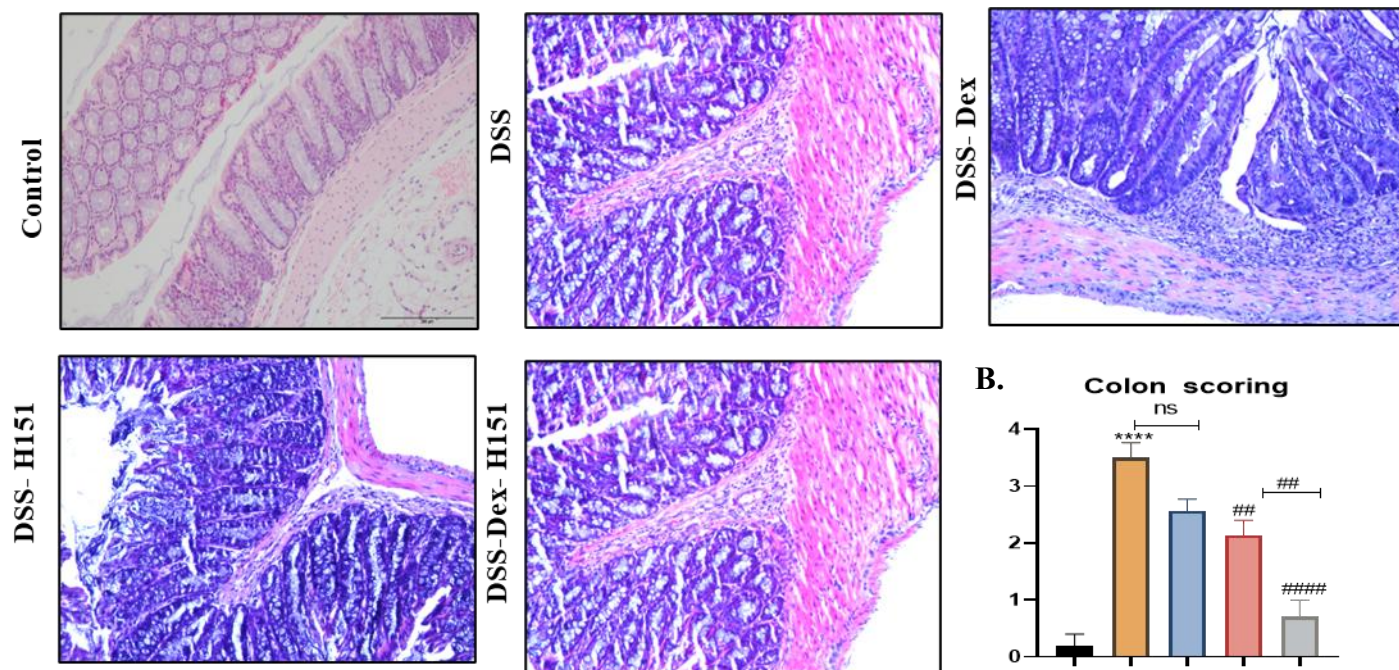

**B.**

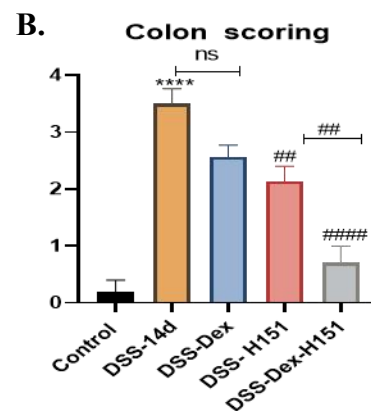

**C.**

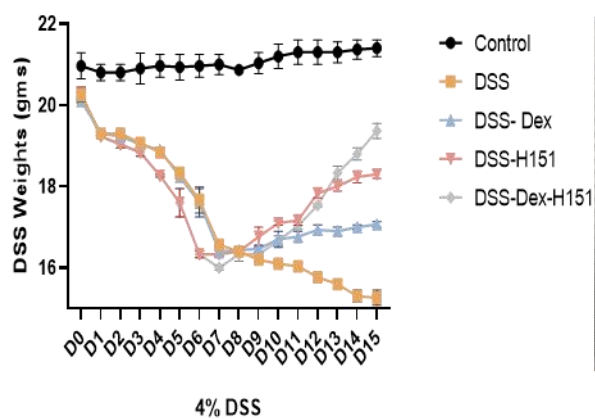

**D.**

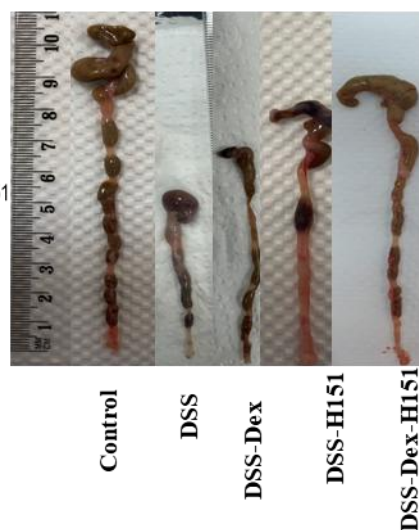

**E.**

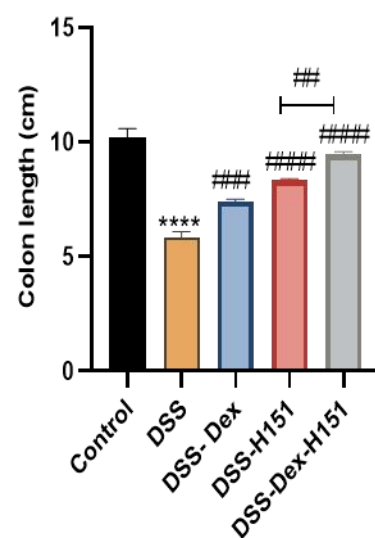

Supplement: S2 Fig — Mice received 4% DSS in drinking water for 7 days, followed by treatment with the STING inhibitor H151, dexamethasone or combination on days 9, 11, and 13. (A-B) Representative H&E-stained colon sections demonstrating severe colitis in DSS-treated mice, partially improved by dexamethasone, markedly attenuated by H151, and most effectively resolved by combined dexamethasone and H151 treatment. (C) Body weight changes expressed as percentage of initial weight. DSS induced progressive weight loss, which was modestly improved by dexamethasone, significantly attenuated by H151, and most effectively prevented by combination therapy. (D) Colon length at sacrifice. DSS caused significant colon shortening; this was partially restored by dexamethasone, more effectively preserved by H151, and maximally restored by combination treatment. Results are presented as mean ± SEM. Statistical analysis was performed using one-way ANOVA followed by a post hoc Bonferroni test for multiple comparisons: *P < 0.05, **P < 0.01, ***P < 0.001, ****P < 0.0001 compared to control mice. #P < 0.05, ##P < 0.01, ###P < 0.001, #### P < 0.0001 compared to DSS mice. (PDF) [file pone.0344511.s002.pdf]

**Supplementary Fig. 3**

**A.**

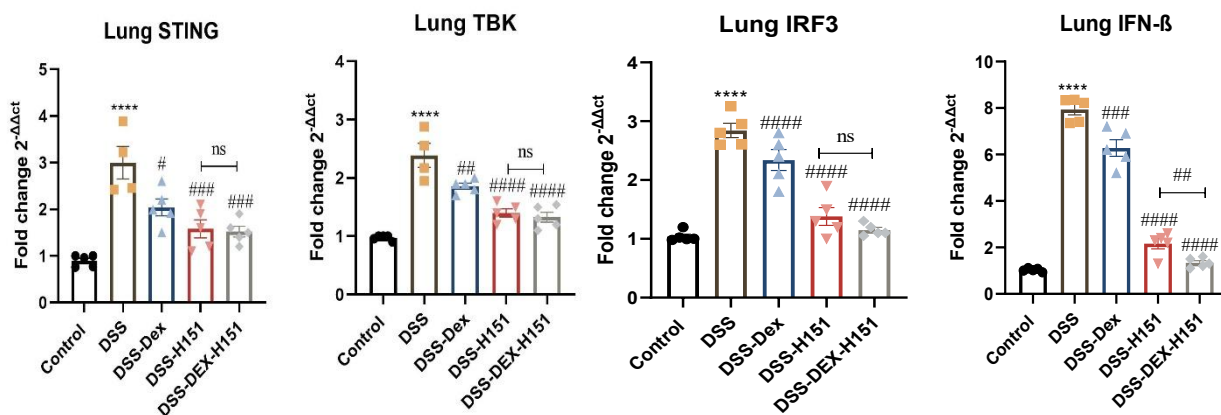

**B.**

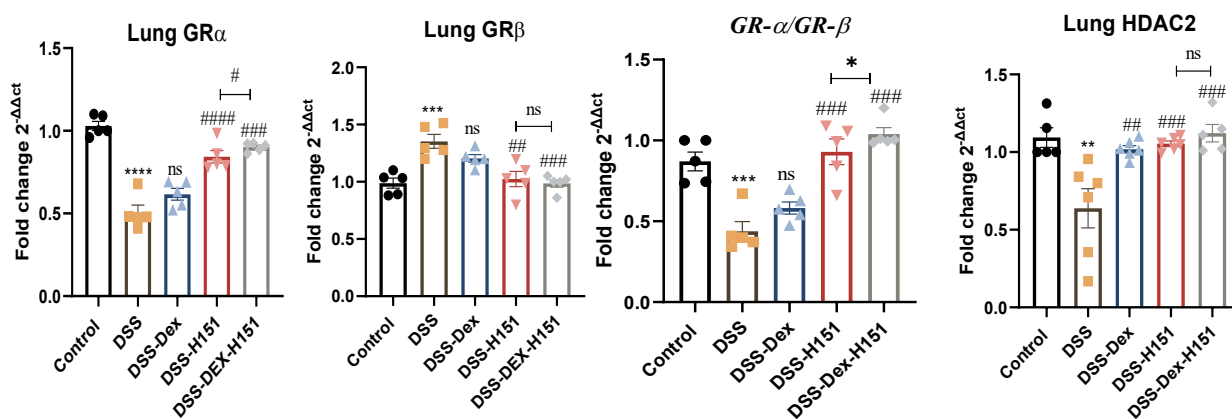

Supplement: S3 Fig — (A) mRNA expression of key STING pathway genes (Sting, Tbk1, Irf3, and Ifn-β) measured by qPCR. H151 significantly downregulated these markers compared to the DSS group, with minor effects from dexamethasone. (B) Representative mRNA expression of GRα, GRβ and HDAC2 in lung tissues. Samples were obtained from control mice and DSS treated mice with or without treatment using Dexamethasone or Sting inhibitor H151 or combination. Data represent n = 5 mice per group. Results are presented as mean ± SEM. Statistical analysis was performed using one-way ANOVA followed by a post hoc Bonferroni test for multiple comparisons: *P < 0.05, **P < 0.01, ***P < 0.001, ****P < 0.0001 compared to control mice. #P < 0.05, ##P < 0.01, ###P < 0.001, #### P < 0.0001 compared to DSS mice. (PDF) [file pone.0344511.s003.pdf]

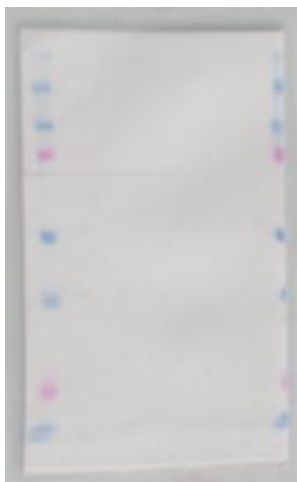

38kd ►

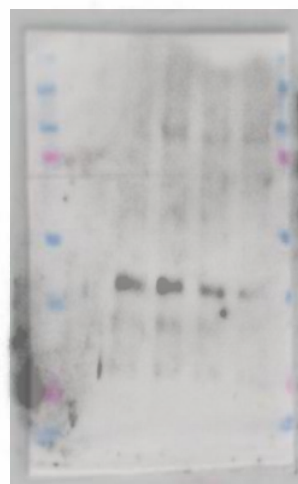

pSTING

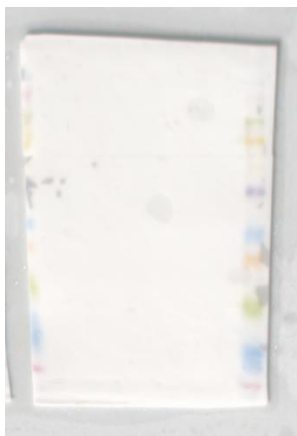

84kd ►

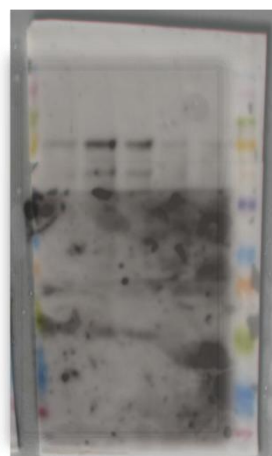

pTBK

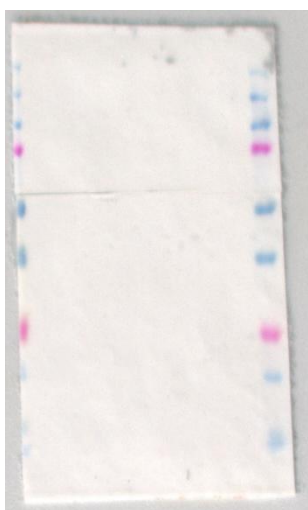

45kd ►

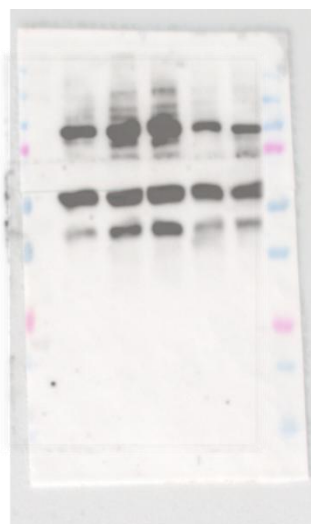

pIRF3

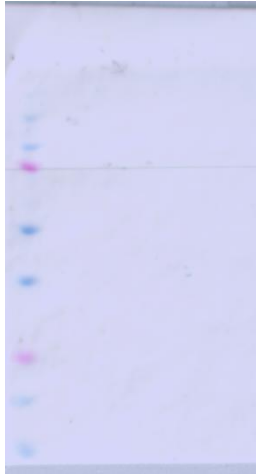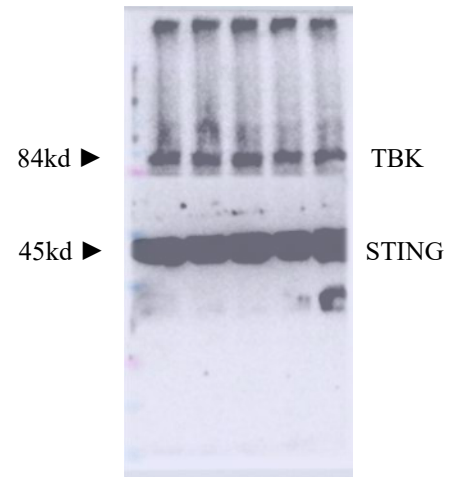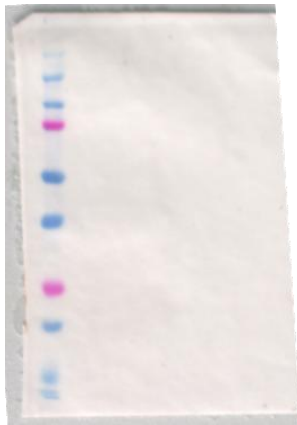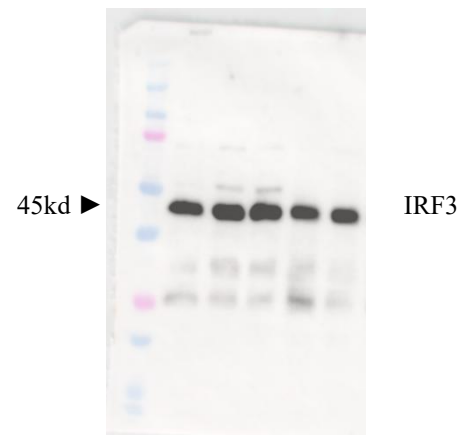

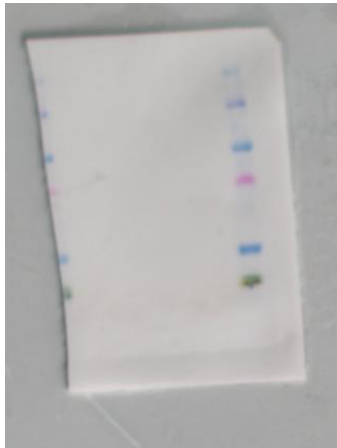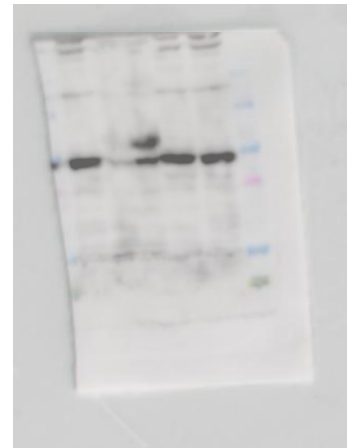

90kd ►

GR $\alpha$

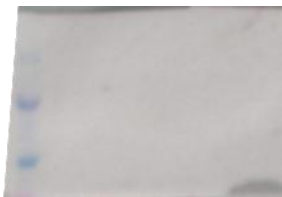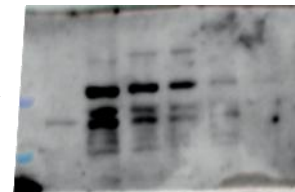

90kd ►

GR $\beta$

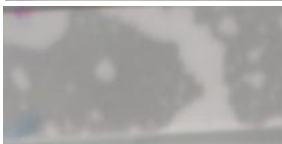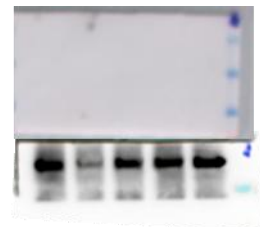

60kd ►

HDAC-2

Supplement: S1 Raw images — (PDF) [file pone.0344511.s004.pdf]
