# Supplementary material for: STING pathway contributes to Steroid-Hyporesponsive Lung Inflammation in DSS-induced colitis mice model
Source: PLoS One. 2026 Mar 27;21(3):e0344511. doi: 10.1371/journal.pone.0344511 (PMC13028359; doi:10.1371/journal.pone.0344511)
Supplement: Attachment — Submitted filename: Response to Reviewers.docx [file pone.0344511.s006.docx]

**Reviewer #1:** Manuscript submission:

In the paper entitled “STING Pathway Contributes to Steroid-Hyporesponsive Lung Inflammation in DSS-Induced Mice Model”, the authors study lung inflammation in a commonly utilized mouse model of inflammatory bowel disease (IBD), that is, the DSS colitis model. They perform experiments to test the hypothesis that bacterial translocation and/or DNA activates the STING pathway, which leads to lung inflammation in this model of disease. They first illustrate the pathologic features of their DSS colitis model and confirm evidence of lung inflammation in the model. They then show that bacterial 16s RNA and RNA and protein of several STING pathway components are upregulated in the lungs of the DSS colitis mice. They then examine biomarkers of steroid responsiveness including GR-a, GR-b, and HDAC-2 in lung tissue, finding a signature of reduced steroid responsiveness in the lungs of DSS colitis mice. Finally, they treat mice with either dexamethasone or H151, a STING pathway inhibitor, and remeasure histologic lung inflammation, STING pathway RNA and protein levels, and the steroid responsiveness biomarkers, overall illustrating that treatment with H151 reduces lung inflammation and increases biomarkers of steroid responsiveness as compared to no treatment. They show that dexamethasone also has this impact, although to a lesser extent, although they do not directly compare dexamethasone treated mice to H151-treated mice, or treat mice with dexamethasone and H151 in succession.

**Response:** We thank the reviewers for their thoughtful and constructive evaluation of our manuscript. We appreciate the clarity with which they summarized our work and the valuable insights provided. Their comments have helped us strengthen the manuscript and refine the presentation of our findings. We are grateful for the time and expertise they devoted to reviewing our study.

**Major comments:**

- Fig 1 (DSS colitis model characterization) could probably be a supplemental figure, or combined with Fig 2, since the DSS colitis model is so well established in the literature. Fig 1 is not offering anything new in this respect. Moreover, much of the data presented in Figures 1 and 2 are replicated in the authors’ recent manuscript published in PLOS One (“The role of gut leakage and immune cell miss-homing on gut dysbiosis-induced lung inflammation in a DSS mice model”: <https://journals.plos.org/plosone/article?id=10.1371/journal.pone.0324230>), providing more impetus for moving these figures to supplemental.

**Response:** We appreciate the reviewer’s suggestion. Figure 1 was moved to the supplementary section (**Supplementary Fig. 1A**).

- Pertaining to the results presented in Figs 4-5:
o Treatment response should also be assessed by looking at established endpoints, similar measures to Fig 1 and 2 (DAI, H&E, weight, colon length etc).

**Response:** We thank the reviewer for this insightful suggestion. We agree that assessing the impact of treatment on gut endpoints would strengthen the study. We have included standard measures such as colon length, body weight, and H&E histology of the colon to illustrate the effects of dexamethasone, H151 and combination on gut inflammation, in (**Supplementary Fig. 2**).

o Rational for looking at these steroid responsive biomarkers should be presented in section 3.4 – could use a shortened version of lines 410-425 (and remove from Discussion)

**Response:** We thank the reviewer for this suggestion. We have included now the rational in section 3.4 line 317-322, highlighting the role of GRα, GRβ, and HDAC2 in glucocorticoid sensitivity.

- Pertaining to results presented in Fig 5:
o DSS-dex and DSS-H151 were compared to DSS alone, but DSS-dex should also be compared to DSS-H151.

**Response:** We thank the reviewer for this comment. In the revised manuscript, we have added this comparison to allow direct evaluation of the relative effects of dexamethasone and H151.

- The authors claim that STING inhibitor restores steroid responsiveness, so they must show that steroids given with STING inhibition improve histologic and protein endpoints. The STING inhibitor could be given in parallel or sequentially with steroids, but this is a key experiment for the central claim of the manuscript.

**Response:** We thank the reviewer for this important and constructive comment. We have now added a new experimental group in which DSS-treated mice received combined dexamethasone and H151 treatment (administered in parallel) in **section 3.5.** **Fig.4**.

o Also, is the GR-a/GR-b ratio also statistically increased in H151 treated mice as compared to DSS and DSS-dex? A plot quantitating the GR-a/GR-b ratio in Figure 5E would help highlight these differences.

**Response:** We thank the reviewer for this valuable suggestion. We have now analysed the GRα/GRβ ratio and included this analysis in **Figure 4E-4F** in the revised manuscript.

- For Supplemental Fig 1A, the authors state that “no noticeable effect” was seen on Sting, Tbk1, Irf3 and IFN-B expression in dexamethasone treated mice; however, all of these had statistically significantly decreased mRNA in dexamethasone treated mice according to p-values presented. The language should be modified, for example “statistically significant but small reduction”.

**Response:** We apologize for the inconsistency. The typo error was corrected in the revised manuscript in section 3.5 line 367.

- Discussion: the authors should not start by stating “This research is the first to demonstrate a direct link between colitis-induced lung inflammation mediated by the STING pathway”. Although the findings they present are suggestive of a connection, they do not directly illustrate that the bacteria translocated from the gut. However, their recent manuscript entitled “The role of gut leakage and immune cell miss-homing on gut dysbiosis-induced lung inflammation in a DSS mice model” (PLOS One) more convincingly illustrates the presence of gut microbes in the lungs of DSS colitis mice. Therefore, the authors should start the discussion by explaining how the findings in their recently published manuscript and the findings in the current manuscript additively support their hypothesis.

- The authors should be careful not to overstate their conclusions. The findings in this manuscript do not prove that the STING pathway was being directly activated by the bacteria. Perhaps using antibiotics or germ-free mice could test this hypothesis, but as the manuscript stands now, a causative link between bacterial translocation and STING pathway activation cannot be established.

**Response:** We thank the reviewer for these insightful comments. We agree that our current data are suggestive but do not directly demonstrate bacterial translocation as the trigger for STING pathway activation. We have revised the Discussion to frame our findings in the context of our previously published work, highlighting how the current results complement and extend our earlier demonstration of gut-derived microbes in the lungs (line 439-448). We have also clearly stated that while the STING pathway is upregulated in this model, a direct link to bacterial translocation remains to be experimentally confirmed (line 465-468).

**Minor comments:**

- Line 64: 28-58% seems high. I looked at the cited literature here and this range is indeed based on 3 very small cohort studies (~30 patients per study). This is not in line with clinical experience. I would suggest removing this statistic and in place saying something like “Once considered rare, IBD-associated pulmonary disease is an increasingly reported entity.”

**Response:** We thank the reviewer for this comment. We now revised the text.

- Lines 140-143: Describe gut and lung inflammation scoring systems (ex, describe the scale 0-4 as shown in Fig 1e and scale 0-3 in 2B) by pathologists in more depth here

**Response:** We thank the reviewer for this comment. We now cited our previously published work in which the same validated scoring criteria were applied (**line 171-172**).

- Line 192: Define “DAI” here (reader might have missed it in Methods, should give reminder)

**Response:** We thank the reviewer for this comment. We now revised the text.

- Lines 253-259: This contains too much conjecture for a Results section. Should be moved to Discussion.

**Response:** We thank the reviewer for this comment. We now revised the text.

- Line 261: is the word “components” missing after “key STING pathway…”?

**Response:** We apologize for the inconsistency. The typo error was corrected in the revised manuscript.

- Typo in line 388, should be “increased lung inflammation”

**Response:** We apologize for the error. The mistake has been corrected in the revised manuscript.

- Line 389: why is gut capitalized?

**Response:** We apologize for the error. The has been corrected in the revised manuscript.

- Lines 405-408 – should these sentences be combined? The sentence starting with “While the NLRP3…” seems incomplete.

**Response:** We thank the reviewer for these comments. We now revised the text.

- Discussion should not reference Figures directly (ex. line 427, line 438, line 441).

**Response:** We thank the reviewer for this suggestion comments. We now revised the text.

- Line 434: should be “triggers”

**Response:** We apologize for the typo. This has been corrected in the revised manuscript.

- Line 436: should be “the STING pathway”

**Response:** We apologize for the error. This has been corrected in the revised manuscript.

**Reviewer #2:** The study of steroid nonresponse and the gut-lung axis in IBD is of interest, and this manuscript attempts to investigate these phenomena through murine DSS colitis model. The authors focus on pulmonary inflammation and changes in response to dexamethasone and H151, a STING (TMEM173) inhibitor.

However, I have several concerns with regards to the methods and how the text is written/presented:

1) The hypothesis/aim(s) is not clear, especially in the context of the methods and results.
- Last paragraph of introduction discusses the importance of studying STING in IBD-associated lung inflammation, and the potential importance of inhibiting STING to restore steroid responsiveness in IBD. The way this paragraph is written, it is not clear if you are interested in: (A) lung inflammation, or (B) if you are interested in bowel disease, or (C) lung inflammation as an EIM of IBD; all in the context of steroid response. If you are interested in IBD, why not study STING and H151 in the colon of DSS-treated mice? And if you are aiming to study the role of STING in the GLA of IBD, then the introduction needs to be more clear on the background literature. Because my understanding is that if IBD is under control (in remission), then in theory there should be no detrimental GLA/pulmonary inflammation. So, the issue is IBD, not pulmonary inflammation.
- My interpretation of the introduction and discussion is that STING inhibition may alleviate steroid hyporesponsiveness/resistance. But you are missing the key experimental group to backup this claim: DSS-treated mice treated with both H151 and dexamethasone. And again, if the background issue (unmet clinical need) is poor response to steroids in IBD patients, then this study needs to be completed in the context of IBD – i.e. the experimental design needs to include gut mucosa.

**Response:** We thank the reviewer for these comments.

We have revised the Introduction and Results sections to more clearly define the study focus and experimental rationale.

In response to the reviewer’s important point regarding steroid responsiveness, we have expanded the experimental design to include a group receiving combined dexamethasone and STING inhibition (DSS + Dex + H151).

2) Paragraph structure:
- the first 1-3 sentences for each of the results sections should either be in the introduction or discussion. Results should be limited to results and not discussion around background literature or implication/interpretation of results
- there are critical methods missing from the methods section. E.g. the dosing schedule of H151 is only listed in a figure caption, but not the methods section.

**Response:** We thank the reviewer for their constructive comments. All the comments were addressed in the revised manuscript.

- Page 3, line 83-85 – this paragraph (or its context) is out of place. This discusses steroid nonresponse in IBD but has nothing to do with the GLA or the study in the paper, which is studying pulmonary inflammation related to IBD and steroid nonresponse in the lungs.

**Response:** We thank the reviewer for this important observation. To address this, we have revised the paragraph to more clearly link steroid hyporesponsiveness in IBD to extraintestinal manifestations, particularly pulmonary involvement, which is the focus of the present study (line 94-100).

3) STING should be more clearly defined/explained in the introduction… e.g. it is a transmembrane protein, encoded by the STING1 gene (also known as TMEM173).

**Response:** We thank the reviewer for this suggestion. We have revised the Introduction to more clearly define STING (line 80-83).

4) Please provide more details on the 16S rRNA in methods.
- was a DNase step completed?
- what controls were implemented to account for contamination? (Especially in context of low biomass tissue, like the lung.)
- in results, 16S is commonly referred in the context of DNA, I assume this is a typo?

**Response:** We thank the reviewer for these important points. We have corrected the terminology throughout the Results and Methods to refer to “Total bacterial 16S” to avoid confusion.

Given the low-biomass nature of lung tissue, appropriate contamination controls were implemented, including negative extraction controls and no-template PCR controls. These details have now been explicitly described in the Methods section 2.6.1 (lines 188-192).

5) How do we know that the intranasal administration of dexamethasone or H151 didn’t have their pulmonary effects, not due to inhalation, but rather due to systemic absorption and/or GI tract ingestion (i.e. pulmonary inflammation was reduced because the DSS colitis improved)?

**Response:** We thank the reviewer for raising this important point, although intranasal administration of dexamethasone and H151 was performed under light sedation by experienced personnel to ensure accurate delivery to the airways, we acknowledge that intranasal dosing cannot completely exclude partial systemic absorption or inadvertent gastrointestinal exposure. Therefore, it is possible that some of the observed pulmonary effects may reflect indirect systemic or gut-mediated effects rather than exclusively local lung actions. This limitation has been acknowledged in the Discussion, and future studies using alternative delivery routes or lung-restricted approaches will be required to fully distinguish local pulmonary effects from systemic contributions (lines 503-507).

6) Can airway responsiveness (AHR) be affected by systemic or intra-abdominal (colonic) inflammation? Severe weight loss? If so, how were these accounted for in the interpretation of your results?

**Response:** We thank the reviewer for raising this important point. We acknowledge that systemic inflammation from colitis and associated weight loss can influence airway hyperresponsiveness (AHR). In our study, body weight was monitored throughout the experiment, and all AHR measurements were interpreted in the context of these changes. While systemic effects cannot be fully excluded, the observed differences in AHR parallel the lung-specific inflammatory and molecular readouts, supporting a direct contribution of pulmonary STING activation to steroid hyporesponsiveness.

7) Page 12 – dexamethasone is described as having no noticeable effect, yet the p values are all below 0.05. Please clarify.

**Response:** We thank the reviewer for pointing this out. We agree that the original wording may be misleading. We have revised the text to indicate that dexamethasone treatment caused a statistically significant difference.

Other comments:
1) Several acronyms are either not defined or are defined on their second use. Please ensure that acronyms are defined upon first use (mostly in introduction). e.g. STING, cGAS, CD, UC.
2) Page 3, Line 83: disagree with word choice “mainstay”. We actively try to avoid steroids, and when used, we limit to the shortest possible course and dose. Mainstay treatments are 5-ASA, biologics, and small molecules (+/- thiopurines).
3) Page 3, Line 58: Use of “chronic” implies there is a different disease, “acute” IBD (but there is no such thing). Remove “chronic”.

**Response:** We thank the reviewer for their comments. All the raised comments were addressed in the revised manuscript.

4) histology scoring was completed by 2 blinded investigators – was there disagreement? How was disagreement resolved?

**Response:** Histological scoring of lung and colon tissues was independently performed by two investigators who were blinded to the experimental groups.

Lines 167-170: “Minor discrepancies in scoring occurred in fewer than 5% of samples and were resolved through joint review and consensus discussion. This approach ensured objectivity, consistency, and reproducibility in the assessment of tissue inflammation and damage.”

5) related to comments above, methods state that both lung and colon tissue were used for gene expression, but it looks like only lung tissue results are presented. Is this correct? Either clarify methods or present colon results as well (if relevant).

**Response:** We thank the reviewer for pointing this out and we apologize for that error. Only lung tissues were used for the gene expression analyses presented in this manuscript. The Methods section has been corrected to reflect this accurately.

**Reviewer #3:**

1.This study innovatively identifies the STING pathway as a core mediator of colitis-induced steroid-hyporesponsive lung inflammation, with a rigorous design, multi-dimensional detection, and confirmation of H151’s superiority over dexamethasone, holding both mechanistic innovation and clinical translational value.

**Response:** We sincerely thank the reviewer for their positive and encouraging comments. We greatly appreciate the recognition of the mechanistic and translational aspects of our study.

2.The study finds H151 restores the GRα/GRβ ratio and HDAC2 expression but fails to explore how the STING pathway specifically regulates these steroid markers, If possible supplement knockdown/overexpression experiments of key STING molecules and detection of inflammatory pathway activity.

**Response:** We thank the reviewer for this valuable suggestion. We agree that mechanistic studies, such as knockdown or overexpression of STING pathway components and assessment of downstream inflammatory signalling, would provide additional insight into how STING regulates GRα/GRβ and HDAC2. While these experiments are beyond the scope of the current study, we have included a statement in the Discussion (lines 486-491), acknowledging this limitation and highlighting it as an important direction for future work.

3.The study lacks specific mouse breeding environment parameters, exact weight monitoring frequency, standardized euthanasia procedures, and should supplement sample size calculation basis or increase repeated experiments to enhance result reliability.

**Response:** We thank the reviewer for these constructive suggestions. We have expanded the Methods section to provide details on the mouse housing and breeding environment, body weight monitoring frequency, and standardized euthanasia procedures. Additionally, we have clarified the basis for our sample size calculations.

4.The study draws conclusions from a mouse model but insufficiently connects to clinical data, requiring supplementary discussion of clinical research progress on the STING pathway in human IBD patients to clarify the link between model conclusions and human diseases.

**Response:** We thank the reviewer for this suggestion. We have added a supplementary paragraph in the Discussion (lines 492-502) highlighting clinical evidence of STING pathway involvement in human IBD.

5.The study meets animal ethics requirements but should supplement mouse distress evaluation criteria in the methods section to improve ethical implementation details.

**Response:** We thank the reviewer for highlighting the importance of distress evaluation. Mouse distress was monitored daily using the Disease Activity Index (DAI), which includes body weight loss, stool consistency, and signs of bleeding, in accordance with institutional ethical guidelines. Any mice reaching humane endpoints were promptly euthanized to ensure ethical compliance.

6.No issues like duplicate publication or conflicts of interest are found, with standardized references and logical data-conclusion consistency, complying with publication ethics.

**Response:** We sincerely thank the reviewer for their positive evaluation and for recognizing the ethical and scientific rigor of our work.

7.This study has significant scientific value, and addressing the above issues through supplementary data and descriptions will improve its completeness and persuasiveness, making it suitable for publication after revisions.

**Response:** We sincerely thank the reviewer for their encouraging comments and constructive suggestions. We have addressed the points raised by providing supplementary data and additional methodological details to improve the completeness and clarity of the manuscript.
