# Supplementary material for: STING pathway contributes to Steroid-Hyporesponsive Lung Inflammation in DSS-induced colitis mice model
Source: PLoS One. 2026 Mar 27;21(3):e0344511. doi: 10.1371/journal.pone.0344511 (PMC13028359; doi:10.1371/journal.pone.0344511)
Supplement: Attachment — Submitted filename: Response_to_Reviewers_auresp_2.docx [file pone.0344511.s007.docx]

**Reviewer #2: Thank you for addressing previous comments/concerns. The paper and its direction is much clearer now.**

I have two remaining questions/concerns:

1) Mice were sacrificed on day 7 or 15, with a total of 25 mice (5 per experimental or control group). There were 5 groups: control, DSS, DSS-Dex, DSS-H151, DSS-Dex-H151. As per figure 1 and 1a, it looks like controls and DSS mice (each N=5) were sacrificed on day 7. But the other groups on day 15? Is this correct? Can you please confirm if you are comparing apples to apples with time courses of treatment and time-lines, clarify as needed.

**Response:** We thank the reviewer for raising this important point, the number of mice were not corrected by mistake. Two independent experimental cohorts were used. In cohort 1, control and DSS mice were sacrificed at day 7 to confirm establishment of lung inflammation. In cohort 2, mice were treated from day 1 to 7 and sacrificed at day 15 to evaluate treatment effects. Comparisons were made within the same timepoint only. We have clarified this in the Methods.

2) Also, it appears that there are no sham/vehicle controls for the Dex and H151 treatment groups - to negate any effects of the vehicle/conditions of treatment (vs the drug of interest itself). Is there any reason not to include a sham group (both for DSS and for intranasal treatments).

**Response:** We apologize for not explicitly clarifying the use of vehicle controls and appreciate the reviewer’s valuable comment.

In the 7-day treatment cohort, control mice received normal drinking water since DSS was given in water. In the 15-day treatment cohort, dexamethasone and H151 were dissolved in PBS and administered intranasally in a total volume of 20 μL per mouse. Control mice in this cohort (15 days) received intranasal PBS (20 μL) following the same schedule as treatment groups. This information has now been clearly detailed in the Methods section.
